# Hypertension: risk perception and health seeking behaviour of long-distance drivers in Port Harcourt

**DOI:** 10.4314/ahs.v23i4.33

**Published:** 2023-12

**Authors:** David Hart, Omosivie Maduka, Amarachi John, Kemdirim Chinonye, Opurum Ndubuisi, Uduak Abiasianam

**Affiliations:** Department of Preventive and Social Medicine, Faculty of Clinical Sciences, University of Port Harcourt, Port Harcourt, Nigeria

**Keywords:** Perception, hypertension, Nigeria, health seeking behaviour

## Abstract

**Background:**

Driving as an occupation is associated with the occurrence of heart-related diseases such as hypertension.

**Objectives:**

To assess the perception of modifiable risk factors of hypertension and the health seeking behaviour of long-distance commercial bus drivers in Port Harcourt Metropolis.

**Methods:**

A cross-sectional study design was used to obtain data from 272 long distance commercial drivers. Data were analysed descriptively using SPSS version 24.0. (*p*≤0.05).

**Results:**

A total of 272 questionnaires were completed and returned (100%). Respondents' mean age was 43.4 ± 8.9. In scoring for perception of modifiable risk factors of hypertension among the drivers, (45.6%) respondents had a high perception score while 148 (54.4%) respondents had a low perception score. In assessing for health seeking behaviour, 190 (69.9%) had checked their blood pressure at least once in their lifetime, 80 (42.1%) of those who had checked, did so over a year ago. However, 18 (9.5%) respondents were on medication for hypertension in the last one month.

**Conclusion:**

This study showed that a greater number of the drivers had a low perception of modifiable risk factors of hypertension. Regular health checks were also not observed among participants. There is need for targeted health education for this group.

## Introduction

Hypertension is a medical condition in which the blood pressure in the blood vessels is constantly or persistently high. It is referred to as a silent killer as it shows little or no symptom in its early stages and is detectable basically by routine blood pressure check 1. Undetected or unmanaged hypertension increases the possibility of developing heart diseases, such as stroke, heart failure and damage to other organs of the human body like the brain and kidneys. Risk factors of hypertension are either modifiable or non-modifiable and according to studies, the prevalence of risk factors is directly responsible for the prevalence of hypertension in adults 2. Modifiable risk factors of hypertension account for over three quarter of cardiovascular diseases and deaths globally 3. It is therefore important and beneficial to make lifestyle modifications to reduce adverse health outcomes of hypertension, particularly in long-distance commercial drivers.

It has been observed that driving as an occupation is associated with the occurrence of heart-related diseases such as hypertension 4, mainly due to exposure to risk factors from the work environment. However, research has shown a low perception of modifiable risk factors of hypertension and poor health seeking behaviour among taxi drivers 5. A study carried out in New York City reported a low perception of the severity of the risk of physical inactivity and unhealthy diet among the taxi drivers 5. Further study conducted in Kenya reported a low perception among the respondents as several of them could not connect behavioural risk factors with the likelihood of developing hypertension 6. Bus drivers often seem to have a poor health-seeking behaviour with routine medical check-up as an indicator 7,8. They barely have time to get routine blood pressure checks partly because they spend their time on the road in transit. Findings from a study in south Brazil observed that though some truck drivers were aware of their hypertensive condition, most of them did not seek regular check-up and were not taking specific medications for their condition 9. Another study in Edo state, Nigeria noted a poor health seeking behaviour among inter-city drivers 7. Regular health checks enable early detection and prompt management of diseases, thereby preventing complications. However, this relevant health behaviour may be altered by high work demands of long-distance commercial drivers.

Aside air travel, road is the major means of travel for most persons in Nigeria. Road transportation fairs are generally less expensive than air transportation and as a direct consequence, a greater population travel by road to and from other parts of Nigeria. Being Africa's most populous nation, Nigeria breeds lots of economic activities, requiring regular inter-city and inter-state movement of persons. This subsequently increases the work demands on the commercial bus drivers who ply long distances, increasing their exposure to risk factors such as sedentary behaviour from driving for long hours, physical inactivity due to busy and erratic work schedules, unhealthy diet due to consumption of processed fast foods or meals high in carbohydrates late at night when they arrive their destination. There is also the likely resort to alcohol and tobacco consumption for their relaxation. This exposure to modifiable risk factors, increases the risk of developing hypertension and other cardiovascular diseases among the drivers.

Although there is increased predisposition of long-distance commercial bus drivers to risk factors of hypertension, not much is known about their perception of modifiable risk factors of hypertension in Nigeria. Since perception of a health condition influences health behaviour and attitudes towards taking up healthy choices 10,11, it is important to assess perception of long-distance drivers in Port Harcourt metropolis for possible strategic interventions. Furthermore, due to the impact of their work demand in Nigeria, there is also the need to assess the drivers' health seeking behaviour as it relates to regular blood pressure check-up and management of hypertensive conditions. The aim of this study, therefore, was to assess the perception of modifiable risk factors of hypertension and the health seeking behaviour among long-distance commercial bus drivers in Port Harcourt Metropolis.

## Methods

### Study area

Port Harcourt Metropolis is situated in Rivers State, it is one of the major urban cities and main economic centers of Nigeria 12. It is in the oil-rich Niger-Delta region of Nigeria, and the Metropolis extends across Port Harcourt Local Government Area and some parts of Obio Akpor and Eleme Local Government Areas and has a population of about 1.85 million inhabitants as at 2016 13. There are two seasons in Port Harcourt Metropolis, dry and rainy season with temperature at a mean maximum of about 34degrees and a mean minimum of about 21degrees 14. Port Harcourt Metropolis is a major industrial hub with several multinational companies, particularly in the petroleum industry, rightly so due to the abundance of natural resources in Rivers State. The tribes commonly found in Port Harcourt city are the Ikwerre, Ijaw, Okrika and Ogoni with English language being the official language. Other languages commonly spoken are Pidgin English and Ibo with majority of the residents as Christians 15.

Most transport companies have their terminals or motor parks in Port Harcourt Metropolis for arrivals and departures. These terminals or motor parks are in clusters distributed in different parts of the Metropolis.

### Study design and study period

A descriptive cross-sectional study design was used to obtain data between 15^th^ of May 2021 and 17^th^ of July 2021.

### Study population

The study population comprised all drivers in all motor parks in Port Harcourt city. Male commercial bus drivers who ply 300km or more on each journey at least 3 times a week, who had been driving professionally for at least 3 years and available at the time of the study were included for the study. Drivers from whom informed consent was not obtained were excluded.

### Sample size determination

The sample size was determined using the sample size formula for descriptive studies 16.

Thus;


$$\begin{array}{c} n=z^{2}\;p.q\\ d^{2} \end{array}$$

Where n is the sample size, z is the coefficient at 95% confidence interval (1.96), p is the population proportion (21.4% from another study)7, q is 1-p and d are the acceptable error (0.05). The calculated minimum sample size was 256 but increased to 282 to accommodate a 10% potential non-response.

### Sampling procedure

A multi-stage cluster sampling technique was used; commercial transport companies who ply long distances exist in six clusters in Port Harcourt Metropolis, a random sampling by ballot was done to select four clusters in the first stage. In the second stage, a total of 24 transport companies were randomly selected from the four cluster. The total number of drivers employed in each selected transport company was then used to calculate a proportionate to size allocation to determine the number of drivers needed from each selected transport company in each cluster. A simple random sampling by balloting was then used to select bus drivers from each selected transport company with the list of drivers provided by the company until the required number allocated to each transport company was obtained.

### Study instrument and data collection

An adapted interviewer administered questionnaire was used 17. The items in the instrument were critically examined for face and content validity. Clarity and appropriateness of language and expression were ensured by pretesting the study instrument among 15 respondents who were not included in the study. A Cronbach's Alpha reliability statistics was further used to determine the reliability of the study instrument. Data were collected on socio-demographic characteristics of the respondents and their perception of modifiable risk factors of hypertension; smoking (tobacco consumption), excessive alcohol consumption (defined as the consumption of more than 4 alcoholic drinks per day or more than 14 alcoholic drinks in one week 18.), excessive salt consumption (≥ 5grams/day), physical inactivity (lack of exercise) and obesity. Participants were asked to indicate their level of agreement or opinion using a three (3) point Likert Scale. Fixed values were assigned from agree=3, neutral=2, disagree= 1. A perception scoring system was obtained by scoring the perception questions. Correctly answered questions were scored 1 and wrongly answered questions were scored 0. The total perception score to modifiable risk factors of hypertension were divided into two; low perception score (0-3) and high perception score (4-7). Data was also collected on health seeking behaviour of the drivers with blood pressure check and last blood pressure check as an indicator.

### Data analysis

Collected data were entered into Microsoft Office Excel 2013 version package for cleaning and management. The data were then exported and analysed using version 24.0 of the Statistical Package for Social Sciences (SPSS). Descriptive statistics (mean, frequency, standard deviation, and percentages) were used to present data.

### Ethical statement

Ethical approval for this study was sought and granted by the Research Ethics Committee of the University of Port Harcourt with reference number: UPH/CEREMAD/REC/MM72/062. Permission was also sought and obtained from the General Managers of the various transport companies chosen for the study. Informed and written consent were obtained from participants before commencement of data collection for the study.

## Results

A total of 272 respondents provided complete data, and the questionnaire had a reliability of 0.8. The mean age was 43.4+8.9, age range 41 to 50 years had the highest frequency. A greater number of the respondents had secondary level education (66.9%, n=182), and a greater number were married (87.5%, n=238). More than three quarter of the respondents have been driving for over 10 years (76.5%, n=208). (Table 1)

**Table 1:** Socio-demographic characteristics of the drivers

Variable	Frequency (N=272)	Percent
**Age (years)**		
21-30	17	6.3
31-40	86	31.6
41-50	120	44.1
51-60	35	12.9
>60	14	5.1
Mean age (years)	43.4(±8.9)	
**Highest level of education**		
Primary	61	22.4
Secondary	182	66.9
Tertiary	29	10.7
**Marital status**		
Single	33	12.1
Married	238	87.5
Divorced/Separated	1	0.4
**Number of years driving**		
≤5 years	14	5.1
6-10 years	50	18.4
Over 10 years	208	76.5
Mean number of years driving	17.7(±8.3)	

Perception of modifiable risk factors of hypertension among the drivers showed that 94 (34.6%) reported that smoking can cause hypertension, 112 (41.2%) reported that excessive alcohol consumption can cause hypertension, 106 (39.0%) reported that too much salt intake can cause hypertension, 119 (43.8%) reported that lack of exercise can cause hypertension, while 101 (37.1%) reported that obesity can cause hypertension. In scoring for perception of modifiable risk factors of hypertension among the drivers, (45.6%) respondents had a high perception score while 148 (54.4%) respondents had a low perception score. (Table 2).

**Table 2:** Perception of modifiable risk factors of hypertension among the drivers

Variables	Frequency (N=272)	Percent
**Smoking can cause Hypertension**		
Agree	94	34.6
Neutral	132	48.5
Disagree	46	16.9
**Excessive alcohol consumption can cause Hypertension**		
Agree	112	41.2
Neutral	128	47.0
Disagree	32	11.8
**Too much salt intake can cause Hypertension**		
Agree	106	39.0
Neutral	135	49.6
Disagree	31	11.4
**Lack of exercise can cause Hypertension**		
Agree	119	43.8
Neutral	108	39.7
Disagree	45	16.5
**Obesity can cause Hypertension**		
Agree	101	37.1
Neutral	143	52.6
Disagree	28	10.3
**Overall Perception score**		
High	124	45.6
Low	148	54.4

Assessing for health seeking behaviour, 190 (69.9%) had checked their blood pressure at least once in their lifetime, 80 (42.1%) of those who had checked, did so over a year ago. 18 (9.5%) respondents reported to be on medication for hypertension in the last one month. (Table 3)

**Table 3:** Health seeking behaviour among the drivers (routine blood pressure check)

Variables	Frequency(N=272)	Percent
**Ever checked blood pressure**		
Yes	190	69.9
No	82	30.1
**last blood pressure check (n=190)**		
1 to 3 months ago	69	36.3
>3 to 6 months ago	22	11.6
>6 to 12 months ago	19	10
Over a year ago	80	42.1

The responses to questions on health-related modifiable lifestyle showed that 207 (76.1%) participants did not smoke any form of tobacco while 65 (23.9%) of the participants still smoked tobacco as at the time of the study. One hundred and eighty-two (66.9%) participants had consumed alcohol in the past, while 132 (48.5%) consumed alcohol regularly. It was also found that 160 (58.8%) participants consumed inadequate amounts of fruits while 139 (51.1%) consumed inadequate amounts of vegetables. Fifty-four (19.9%) participants consumed inadequate amounts of salt. Sedentary behaviour was also assessed. It was noted that 137 (50.4%) spent 9 hours or more driving. More than half (57.0%, n=155) of the participants did not perform any form of moderate/vigorous-intensity activities. Fifty (4%) of the participants reported a family history of hypertension. See Table 4.

**Table 4:** Health-related modifiable lifestyle of study participants

Variable	Frequency N = 272	Percentage
**Currently smoke tobacco**		
Yes	65	23.9
No	207	76.1
**Smoke tobacco daily**		
Yes	34	12.5
No	238	87.5
**Duration of smoking**		
1 - 3	24	8.8
>3 to <6 year	8	2.9
6 years and above	33	12.1
**Currently consume alcohol**		
Yes	182	66.9
No	90	33.1
**Regular alcohol consumption (at least 4 days per week**		
Yes	132	48.5
No	140	61.5
**Salt consumption**		
Inadequate	54	19.9
Adequate	218	80.1
**Fruit consumption**		
Inadequate	160	58.8
Adequate	112	41.2
**Vegetable consumption**		
Inadequate	139	51.1
Adequate	133	48.9

**Number of hours spent driving in a day**		
1-2 hours	62	22.8
3-4 hours	73	26.8
5 hours or more	137	50.4

## Discussion

In assessing for perception of modifiable risk factors of hypertension among long distance commercial bus drivers in Port Harcourt Metropolis, this study revealed a low perception score in a greater population of the drivers, like findings in New York City 5 which reported a low perception to the severity of physical inactivity and unhealthy diet. The current findings are also in agreement with findings in Nairobi, Kenya 6 which reported a low perception of modifiable risk factors among commercial drivers. This seemingly low perception among a greater population of the drivers may be attributed to their level of education as it was observed that a greater number of the drivers had secondary school education as their highest level of education. This is not surprising as educational qualification is not a prerequisite to engage in professional driving in Nigeria. The potential under-estimation of the adverse health outcomes of undetected or unmanaged hypertension due to a low perception may contribute to a rise in the prevalence of hypertension among this occupational group. This is possible because of the risk factors these drivers are exposed to in their work environment and the lack of lifestyle modification to address these risk factors.

This study also reported a poor health seeking behaviour using routine blood pressure check-ups as an indicator. More than one quarter of the drivers had never checked their blood pressure in their lifetime. This is consistent with findings in Edo state, Nigeria which reported that over half of those who claimed to have checked their blood pressure sparsely did so 7. Findings from south Brazil also showed that though some truck drivers were aware of their hypertensive condition, most of them did not seek regular check-ups and were not taking specific medications for their condition 9. It is noteworthy that poor health seeking behaviour is not peculiar to long distance bus drivers alone 19. The poor health seeking behaviour with medical checks as an indicator, might be due to ignorance, poor perception of hypertension or the busy work schedule of these drivers, as they tend to spend a greater part of the day and sometimes night in transit. This seemingly lack of routine blood pressure check could be a precursor to the onset of the adverse health effects of undetected or untreated hypertension such as vision impairment, heart attack, stroke, and kidney failure among this occupational group. This could increase the chances of early retirement from work, potential risk for accidents, loss of life in the event of an accident not just for the drivers, but also for the commuting public. The cost of repairing or replacing damaged vehicles due to accidents could also lead to economic losses for the transport companies.

High prevalence of unhealthy modifiable lifestyle was also observed in this study. In another study, professional male drivers in Lagos, Nigeria, had a high prevalence of cluster risk factors for CVDs, with a high proportion of drivers using alcohol 22. A history of smoking was also an independent risk factor for hypertension among drivers in Ghana 23. Unhealthy modifiable lifestyle is associated with several chronic diseases 20. There is a direct relationship between excessive alcohol consumption and the risk of developing CVDs. Harmful alcohol use has been reported to cause damage to the muscles of the heart and increases the risk of stroke and other CVDs 21. Both unhealthy diet and obesity have also been linked with an increased risk of developing cardiovascular diseases 21.

This study has enhanced understanding of long-distance commercial drivers' perception of hypertension, and has also contributed to the body of evidence on the need for necessary interventions for these occupation group. However, limitations of recall bias may be associated with the study.

## Conclusion

As studies continue to link the occupation of driving with the likelihood of developing hypertension and other cardiovascular diseases due to exposure to predisposing factors, this study showed that a greater number of the drivers had a low perception of modifiable risk factors of hypertension. Regular health checks were also not observed among most participants. There is the need for adequate health education programs on hypertension and other heart-related diseases targeting this occupational group to help change their perception, hopefully their behavioural lifestyle and lead to positive health outcomes. There is also the need to ensure that all long-distance transport companies in Port Harcourt Metropolis, establish a partnership with a Health Maintenance Organisation (HMO) to provide routine blood pressure checks, counselling and other medical services for the drivers. This will ensure regular monitoring of their blood pressure, effectively manage hypertension, and detect any emergencies that may arise to offer prompt medical care.

## References

[1] World Health Organization (2015). A global brief on Hypertension - Silent Killer, Global Public Health Crisis. Women.

[2] World Health Organization (2021). WHO |Hypertension factsheet.

[3] Yusuf S, Joseph P, Rangarajan S, Islam S, Mente A, Hystad P, Brauer M, Kutty VR, Gupta R, Wielgosz A, AlHabib KF (2020). Modifiable risk factors, cardiovascular disease, and mortality in 155 722 individuals from 21 high-income, middle-income, and low-income countries (PURE): a prospective cohort study. The Lancet.

[4] Reis LAP, dos Costa CDD, Rodrigues DS, Alcântara KC de (2017). Obesity, hypertension and diabetes among truck drivers in the middle-west, Brazil. Bioscience Journal.

[5] Ukudeyeva A, Ramirez LR, Rivera-Castro A, Faiz M, Espejo M, Kanna B (2018). 2460 Qualitative study of obesity risk perception, knowledge, and behavior among Hispanic taxi drivers in New York. Journal of Clinical and Translational Science.

[6] Wekesah FM, Kyobutungi C, Grobbee DE, Klipstein-Grobusch K (2019). Understanding of and perceptions towards cardiovascular diseases and their risk factors: a qualitative study among residents of urban informal settings in Nairobi. BMJ open.

[7] Tobin EA, Ofili AN, Asogun DA, Igbinosun PO, Igba KO, Idahosa AV (2013). Prevalence of hypertension and associated factors among inter-city drivers in an urban city in South-South Nigeria. International Journal of Research in Medicine.

[8] Erhiano EE, Igbokwe VU, El-khashab MM, Okolo RU, Awosan KJ (2015). Prevalence of hypertension among commercial bus drivers in Sokoto, Sokoto State, Nigeria. Journal of Medicine and Medical Sciences.

[9] Sangaleti CT, Trincaus MR, Baratieri T, Zarowy K, Ladika MB, Menon MU (2014). Prevalence of cardiovascular risk factors among truck drivers in the South of Brazil. BMC Public Health.

[10] Cendales-Ayala B, Useche SA, Gómez-Ortiz V, Bocarejo JP (2017). Bus operators' responses to job strain: An experimental test of the job demand–control model. Journal of occupational health psychology.

[11] Kolb SE, Zarate-Abbott PR, Gillespie M, Deliganis J, Norgan GH (2011). Perceptions about high blood pressure among Mexican American adults diagnosed with hypertension. Family & community health.

[12] Akukwe TI, Ogbodo C (2015). Spatial analysis of vulnerability to flooding in Port Harcourt metropolis, Nigeria. Sage Open.

[13] Demographia (2016). Demographia World Urban Areas.

[14] Uko ED, Tamunobereton-Ari I (2013). Variability of climatic parameters in Port Harcourt, Nigeria. Journal of Emerging Trends in Engineering and Applied Sciences.

[15] Kari EE (2002). Multilingualism in Nigeria: The example of Rivers State. In a Paper Presented at the Seminar on Multilingual Situation and Related Local Cultures in Asia and Africa Institute for the Study of Languages and Cultures of Asia and Africa.

[16] Singh AS, Masuku MB (2014). Sampling techniques & determination of sample size in applied statistics research: An overview. International Journal of economics, commerce and management.

[17] Seham A, Samira E (2015). Knowledge and perceptions related to hypertension, lifestyle behavior modifications and challenges that facing hypertensive patients. IOSR Journal of Nursing and Health Science.

[18] National Institute of Alcohol Abuse and Alcoholism Drinking level defined.

[19] Eze UIH, Osoba DO, Iheanacho CO (2021). Non-diabetic Out-Patients' Lifestyle and Awareness of Type 2 Diabetes Symptoms in two Nigerian Secondary Health Care Facilities. International Journal of Human and Health Sciences.

[20] Iheanacho CO, Osoba DO, Eze UIH (2021). Evaluation of predominant risk factors for type-2 diabetes mellitus among out-paints in two Nigerian secondary health facilities. African Health Sciences.

[21] Mendis S, Puska P, Norrving B (2011). Global atlas on cardiovascular disease prevention and control.

[22] Amadi C E, Grove TP, Mbakwem AC, Ozoh OB, Kushimo OA, Wood DA, Akinkunmi M (2018). Prevalence of cardiometabolic risk factors among professional male long-distance bus drivers in Lagos, south-west Nigeria: A cross-sectional study. Cardiovascular Journal of Africa.

[23] Anto EO, Owiredu WKBA, Adua E, Obirikorang C, Fondjo LA, AnnaniAkollor ME (2020). Prevalence and lifestyle-related risk factors of obesity and unrecognized hypertension among bus drivers in Ghana. Heliyon.

